# Capturing Chemotherapy and Radiotherapy Dose Among Breast Cancer Patients With the Utah All‐Payer Claims Database Compared With Gold‐Standard Abstraction

**DOI:** 10.1002/cam4.70411

**Published:** 2024-11-15

**Authors:** Alzina Koric, Chun‐Pin Esther Chang, Shane Lloyd, Mark Dodson, Vikrant G. Deshmukh, Michael G. Newman, Ankita P. Date, Jen A. Doherty, Lisa H. Gren, Christina A. Porucznik, Benjamin A. Haaland, N. Lynn Henry, Mia Hashibe

**Affiliations:** ^1^ Division of Public Health Sciences, Department of Surgery Washington University School of Medicine St. Louis Missouri USA; ^2^ Huntsman Cancer Institute Salt Lake City Utah USA; ^3^ Division of Public Health University of Utah School of Medicine Salt Lake City Utah USA; ^4^ University of Utah School of Medicine Salt Lake City Utah USA; ^5^ Intermountain Healthcare Salt Lake City Utah USA; ^6^ University of Utah Health Sciences Center Salt Lake City Utah USA; ^7^ Pedigree and Population Resource, Population Sciences Huntsman Cancer Institute Salt Lake City Utah USA; ^8^ Utah Cancer Registry University of Utah Salt Lake City Utah USA; ^9^ Department of Population Health Sciences University of Utah School of Medicine Salt Lake City Utah USA; ^10^ Division of Hematology and Oncology, Department of Internal Medicine University of Michigan Medical School Ann Arbor Michigan USA

**Keywords:** APCD, breast cancer, chemotherapy, gold standard, methods, radiotherapy dose

## Abstract

**Objective:**

To evaluate the validity of the Utah statewide All‐Payer Claims Database (APCD), we compared breast cancer‐specific treatments and dosages with gold‐standard abstraction of medical records.

**Study Design:**

In this pilot study, breast cancer treatments were abstracted by a certified tumor registrar at the Utah Cancer Registry (UCR) for patients diagnosed in 2013 with breast cancer. The abstraction of medical records was the *gold standard* for comparison with treatments identified in the APCD. The reliability and agreement between the treatment identified in the APCD and abstraction data were measured with sensitivity and specificity. Dose consistency was measured with the intraclass correlation coefficients (ICC).

**Results:**

Compared with the 186 abstractions, the sensitivity of the APCD to identify chemotherapy agents was high: 89% for any agent, 91% for carboplatin, 83% for docetaxel, 82% for doxorubicin, or 94.7% for biologic therapy. The consistency between the chemotherapy dosage identified in the claims and the abstraction varied from 63% to 76%. For radiotherapy, the sensitivity of the claims to identify the completed radiotherapy regimen was 66%. The ICC between radiotherapy doses identified in the claims and the abstraction was 54% (95% confidence interval [CI], 48%, 67%).

**Conclusions:**

Employing these novel methods, the claims were highly reliable in identifying cancer treatment agents overall, namely carboplatin, docetaxel, and trastuzumab. The claims were of moderate utility in capturing the treatment dose information. In addition to the APCD, the use of multiple data sources improved the completeness of cancer treatment information.

## 1 Introduction

As the number of breast cancer survivors in the United States (US) is expected to surpass 4 million by 2026, [1] understanding the effects of breast cancer treatments related to survival becomes increasingly important. The National Cancer Institute's (NCI) Surveillance, Epidemiology, and End Results (SEER) population‐based cancer registries collect information on first‐course cancer‐related treatments for a large proportion of the US population [2, 3]. However, due to known cancer treatment incompleteness across registries, the NCI does not provide chemotherapy agent or dose data for research. SEER has been shown to be more beneficial for research when augmented with Medicare, particularly in relation to chemotherapy use [4] or the completeness of radiotherapy [5, 6]. Private payer claims were not included in these SEER Medicare‐specific studies, and details such as breast cancer chemotherapy or radiotherapy dose and treatment regimen names were not assessed.

For research purposes, data sources such as the All‐Payer Claims Database (APCD) can be utilized to identify breast cancer treatment completeness. APCDs include medical, pharmacy, and dental claims collected from private and public payers [7]. The Utah Department of Health, one of the 18 states with an established APCD, covers about 90% of patients with commercial health insurance plans, aligning with the American Community Survey (ACS) estimates for uninsured Utahns at 9.7% in 2020 [8, 9, 10]. The validity of APCDs to identify detailed breast cancer treatment information has not been established. We developed methods to capture breast cancer chemotherapy regimens and dosages, as well as radiotherapy completion and dose information in APCD. We compared our methods to (gold standard) data abstracted by a certified tumor registrar.

## 2 Methods

### 2.1 Data Sources

The Utah Cancer Registry (UCR) is among the seven original National Cancer Institute's (NCI) SEER registries [11]. The UCR is linked to the Utah Population Database (UPDB), which contains electronic medical records (EMRs), the statewide healthcare facility database (SHFD), and patient demographic information. For this study, cancer treatments were abstracted for 197 breast cancer patients ages ≤ 65 at breast cancer diagnosis in 2013. Following the inclusion of only Utah‐based claims, 186 patients were included in the analysis. The cancer treatment details were manually abstracted from medical records by a certified tumor registrar (CTR) at the UCR, using the NCI Patterns of Care method [12]. The abstracted records were used as a gold standard for comparison with treatments identified in APCD. A subanalysis was conducted to assess the agreement between treatment information identified in other data sources available in UPDB including EMRs, SHFD, and pharmacy and medical claims provided by UCR, with the abstracted data.

### 2.2 Treatment Identification

The APCD pharmacy records were used to identify treatment information based on the National Drug Code (NDC) for endocrine therapy, and medical claims based on the Healthcare Common Procedure Coding Systems (HCPCS)/Common Procedural Terminology (CPT) billing codes for identification of chemotherapy agents. The NCI SEER's Cancer Medications Enquiry Database (CanMED) SEER*Rx database [13] was used to obtain the appropriate HCPCS/CPT billing codes and the NDC for the identification of chemotherapy, endocrine therapy, and trastuzumab within the claims. The American Society of Radiation Oncology (ASTRO) list of oncology HCPCS/CPT procedure billing codes was used to identify radiotherapy treatment delivery codes, including 77,401, 77,402, 77,412, 77,413, 77,414, 77,416, 77,418, 77,787, and 77,778, within APCD [14]. The billing codes for radiotherapy treatment planning were not counted toward radiotherapy completion.

### 2.3 Systemic Therapy

Patients with an HCPCS billing code for infusion chemotherapy in the claims were categorized as having received chemotherapy; those without such a code were categorized as not having received chemotherapy. Similarly, patients that had a billing code associated with endocrine therapy (tamoxifen Nolvadex) or aromatase inhibitors (Anastrozole, Letrozole, or Exemestane) or biologic therapy (trastuzumab [Herceptin]) were classified as having received these treatments.

*Chemotherapy cycles*: Each date of service in the claims represented a cycle, including the rest period, which lasted until the next service date, which was counted as the next cycle or otherwise known as a treatment cycle [15]. *Chemotherapy dose*: dose was estimated as commonly recommended by National Comprehensive Cancer Network (NCCN) [16] guidelines for most commonly identified chemotherapy agents including docetaxel (Taxotere), doxorubicin (Adriamycin) cyclophosphamide (Cytoxan), paclitaxel (Taxol), and carboplatin (Paraplatin), as defined in the supporting materials (online Table S1). For carboplatin, the dose was calculated using the Calvert equation [17] with the established target area under the plasma concentration/time curve of 6 (AUC mg/mL/min) when the treatment was given every 21 days, as commonly recommended dosing by the NCCN [16]. The cumulative dose was estimated by multiplying the number of cycles identified in the APCD by the appropriate calculated dose. For example, the recommended NCCN dose for doxorubicin is 60 mg/m^2^; the cumulative dose was estimated by multiplying this dose with the patient's body surface area (BSA), which was then multiplied by the total number of cycles identified for each patient in the APCD and abstraction. BSA was calculated with Mosteller's formula [18].

### 2.4 Radiotherapy

The service dates in the claims for radiotherapy were reviewed to estimate the total number of days of consecutive radiotherapy fractions. *Radiotherapy fractions*: defined as the number of treatments or fractions that are given five consecutive days per week, over several weeks [2]. There are currently three main fractionation schedules; (a) standard fractionation, with a recommended dose per fraction from 1.8 Gray (Gy) to 2.0 Gy for 25 to 28 fractions, potentially followed by a boost, (b) hypofractionated irradiation, with a recommended dose per fraction from 2.5 Gy to 3.0 Gy for 15–16 fractions, and (c) accelerated partial breast irradiation, usually over 3.0 Gy per fraction for 10 fractions delivered twice a day [16]. Radiotherapy dose was not estimated for patients with fewer than 5 fractions identified in the claims.

The total number of radiotherapy fractions was totaled based on the number of single service dates identified as billed in the claims. The number of consecutive fractions since the prior treatment date within a year since the breast cancer diagnosis and with no more than 30 days between treatments were estimates, [19, 20] which would have otherwise been considered an interrupted radiotherapy treatment (Table S2). Our team's oncologists were instrumental in ensuring that all dosing in the data was clinically sound. Within the abstracted data, radiotherapy information included the number of total fractions and total dose in Gray (Gy) received for the initial radiotherapy regimen, and any subsequent radiotherapy boost treatments.

### 2.5 Statistical Analysis

To compare treatment receipt identified from the claims to the abstraction (*gold‐standard*), the Cohen's Kappa statistic (ĸ) was used (measuring agreement between categorical measures, e.g., treatment name [yes/no], correcting for chance agreement): ĸ = P_o_—P_e_/1—P_e_, where P_o_ is the observed concordance (number in agreement/total) and P_e_ is the concordance expected, based on row/columns total. As a proportion, concordance ranges from 0 to 1 (> 0.80 almost perfect agreement). Sensitivity, specificity, positive predictive value (PPV), and negative predictive value (NPV) and 95% confidence intervals (CI) were estimated to evaluate the reliability of the claims against the gold‐standard. For the subanalysis, the agreement statistic was reported for all data sources combined and for each individual data source against the gold standard (95%CI reported online in the supporting material, Tables S3–S5) to show the individual contributions in treatment identification for each additional data source against the abstraction.

To measure the consistency between the continuous variables on the treatment dose, the intraclass correlation coefficient (ICC) was estimated: ICC = σ2B / σ2B + σ2W, (σ2B denotes between data‐ and σ2W within‐data variance, as that total variance = σ2B + σ2W). The ICC value close to 1 denotes high consistency. The ICC calculation was done using the STATA *kappaetc* (default model) function to measure the consistency of dose agreement between the claims and abstraction data. A log‐transformation of the dose was assessed as sensitivity analysis to reduce skewness and better align the range of values [21]. This study was approved by the University of Utah Institutional Review Board (IRB) and the Resource for Genetic and Epidemiologic Research (RGE).

## 3 Results

**FIGURE 1:**
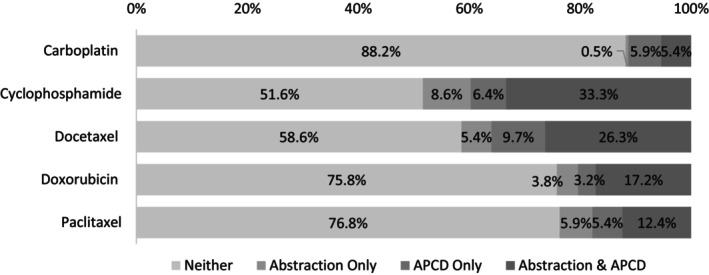
Agreement (%) between chemotherapy agents identified in the Utah All‐Payer Claims Database (APCD) compared with abstraction (gold‐standard), *n* = 186.

Among the 186 abstractions, 43.0% of breast cancer patients were 55 years of age or younger (Table 1). The predominant breast cancer subtype was HR+/HER2‐ (72.0%) and tumors of ductal origin (75.3%). The agreement between chemotherapy agents identified in the abstraction and the claims is shown in Figure 1. For example, 8.6% of patients had cyclophosphamide in the abstraction only, 6.4% in the claims only, 33.3% in the claims and abstraction, and 51.6% in neither. On average, chemotherapy treatment initiation occurred within 3–4 months following breast cancer diagnosis and concluded within a year from the cancer diagnosis.

**TABLE 1:** Characteristics of 186 patients diagnosed with breast cancer in 2013 with treatment abstracted by a certified tumor registrar.

	No.	%
**Age at diagnosis (y)**		
< 45	36	(19.3)
45–54	70	(37.6)
55–64	80	(43.1)
**Race**		
White	176	(94.6)
Other	—^a^	—^a^
**Hispanic**		
Yes	21	(11.3)
No	165	(88.7)
**Histology**		
Ductal	140	(75.3)
Lobular	33	(17.7)
Other	13	(7.0)
**Cancer grade**		
Grade I	43	(23.1)
Grade II	86	(46.2)
Grade III	53	(28.5)
Unknown	—^a^	—^a^
**Cancer subtype**		
HR+/HER2−	134	(72.0)
HR+/HER2+	18	(9.7)
HR−/HER2+	—^a^	—^a^
HR−/HER2−	18	(9.7)
Unknown	—^a^	—^a^
**AJCC stage**		
Stage I	80	(43.0)
Stage II	67	(36.0)
Stage III–IV	35	(18.8)
Unknown	—^a^	—^a^
**Surgery type**		
None	12	(5.9)
Lumpectomy	94	(50.6)
Mastectomy	80	(43.5)
**Health insurance coverage**		
Self‐pay	—^a^	—^a^
Private insurance	138	(74.2)
Medicaid	26	(14.0)
Medicare/Medicaid, NOS	—^a^	—^a^
Other^**b**^	—^a^	—^a^

Abbreviations: AJCC, American Joint Committee on Cancer; HR, hormone receptor; HER, human epidermal growth factor receptor; NOS, not otherwise specified.

^**a**^ Suppressed if the number of observations was < 11 in accordance with the data confidentiality policy.

^**b**^ Other health insurance coverage included Military TRICARE Health Insurance and Indian/Public Health Services/NOS.

The sensitivity of the APCD to identify specific chemotherapy treatment agents varied, with high sensitivity overall for any chemotherapy (89.4%). PPV was high for any chemotherapy (93.9%), indicating that among patients identified by the abstractor as receiving any chemotherapy, a high proportion also had the claims (Table 2). The reliability of the APCD to capture endocrine or biologic therapy was demonstrated with a sensitivity of 81.2% for tamoxifen, 81.0% for aromatase inhibitors, and 94.7% for trastuzumab. For each data source assessed individually (EMRs, SHFD, and additional one‐time pharmacy and medical claims provided by UCR), except for APCD alone, the overall sensitivity and agreement were low for identifying treatment agent names (Table 3). With the addition of all data sources to the APCD, sensitivity increased from 89.4% to 96.1% for any chemotherapy, 67.0%–73.5% for paclitaxel, 82.0%–89.7% for doxorubicin, 79.5%–87.1% for cyclophosphamide, and from 83.0% to 88.1% for docetaxel (Table 3).

**TABLE 2:** Comparison of cancer treatment information identified in All‐Payer Claims Database (APCD)^a^ and abstraction (gold standard) among 186 patients diagnosed with breast cancer in 2013.

Treatment agents	Neither abstraction nor APCD		Abstraction only		APCD only		Both abstraction and APCD		ĸ		Sensitivity		Specificity		Positive predictive value		Negative predictive value	
	*N* (row %)								%	(95% CI)	%	(95% CI)	%	(95% CI)	%	(95% CI)	%	(95% CI)
Chemotherapy, any^b^	75	(40.3)	—^**c**^	—^**c**^	—^**c**^	—^**c**^	93	(50.0)	80.5	(71.9, 89.0)	89.4	(81.9, 94.6)	91.5	(83.2, 96.5)	93.0	(86.1, 97.1)	87.2	(78.3, 93.4)
**Chemotherapy agents**																		
Carboplatin	164	(88.2)	—^**c**^	—^**c**^	—^**c**^	—^**c**^	10	(5.4)	59.3	(38.8, 79.8)	91.0	(73.9, 93.0)	93.7	(90.1, 97.3)	48.0	(26.3, 68.9)	99.0	(98.0, 1.00)
Cyclophosphamide	96	(51.6)	16	(8.6)	12	(6.4)	62	(33.3)	69.0	(58.3, 79.5)	79.5	(70.5, 88.4)	88.9	(83.0, 95.0)	83.8	(75.4, 92.2)	85.7	(79.3, 92.2)
Docetaxel	109	(58.6)	—^**c**^	—^**c**^	18	(9.7)	49	(26.3)	66.5	(55.1, 77.8)	83.0	(73.5, 92.6)	85.8	(79.8, 91.9)	73.1	(62.5, 83.7)	91.6	(86.6, 96.6)
Doxorubicin	141	(75.8)	—^**c**^	—^**c**^	—^**c**^	—^**c**^	32	(17.2)	79.0	(67.6, 89.8)	82.5	(70.0, 94.0)	95.9	(92.7, 99.0)	84.2	(72.6, 95.8)	95.3	(91.8, 97.8)
Paclitaxel	142	(76.8)	—^**c**^	—^**c**^	—^**c**^	—^**c**^	23	(12.4)	62.0	(47.0, 76.6)	67.6	(52.0, 83.4)	93.4	(89.5, 97.4)	69.7	(54.0, 85.4)	92.8	(88.7, 96.9)
**Endocrine therapy**																		
Tamoxifen	115	(61.8)	—^**c**^	—^**c**^	25	(13.4)	39	(21.0)	60.0	(46.4, 71.5)	81.2	(70.0, 92.3)	83.3	(77.1, 89.5)	62.9	(51.0, 75.0)	92.7	(88.2, 97.3)
Aromatase inhibitors^d^	83	(44.6)	13	(7.0)	36	(19.3)	54	(29.0)	47.0	(34.5, 59.2)	81.0	(71.0, 90.0)	69.7	(61.5, 78.0)	60.1	(50.1, 70.0)	86.5	(79.6, 93.3)
**Biologic therapy**																		
Trastuzumab	160	(86.0)	—^**c**^	—^**c**^	—^**c**^	—^**c**^	18	(9.87)	79.4	(65.7, 93.1)	94.7	(84.7, 1.00)	95.8	(92.8, 98.8)	72.0	(54.0, 89.6)	99.4	(98.2, 1.00)

Abbreviations: ĸ, kappa statistic (values from 0.41 to 0.60 = moderate agreement, 0.61 to 80 = substantial agreement, and ≥ 0.81 = perfect agreement); CI, confidence interval.

^a^ APCD was combined with pharmacy and medical claims provided by the Utah Cancer Registry (UCR).

^b^ Any chemotherapy indicates having received at least one chemotherapy agent.

^c^ Suppressed if the number of observations was < 11 in accordance with the data confidentiality policy.

^d^ Aromatase inhibitors (AIs) included anastrozole, letrozole, or exemestane. One patient for carboplatin and two patients for doxorubicin had these treatments beyond the first course (postprimary treatment was not included in this analysis).

**TABLE 3:** Comparison of cancer treatment information identified in individual data sources, all sources combined ^a^ and abstraction (gold‐standard) among 186 patients diagnosed with breast cancer in 2013.

	Source																								
	EMR only					Pharmacy and medical claims from UCR only					APCD only					APCD + pharmacy and medical claims from UCR					All Sources^a^				
Treatment agents	ĸ	SE	SP	PPV	NPV	ĸ	SE	SP	PPV	NPV	ĸ	SE	SP	PPV	NPV	ĸ	SE	SP	PPV	NPV	ĸ	SE	SP	PPV	NPV
	%	%	%	%	%	%	%	%	%	%	%	%	%	%	%	%	%	%	%	%	%	%	%	%	%
Chemotherapy, any^**b**^	24.5	29.1	97.6	93.7	52.3	26.0	30.8	97.6	94.0	52.6	81.3	89.4	92.5	93.9	87.1	80.5	89.4	91	93.0	87.2	87.0	96.1	90.2	92.6	94.9
**Chemotherapy agents**																									
Carboplatin	51.8	36.4	100.0	100.0	96.1	28.0	27.3	97.1	37.5	95.5	59.3	91.0	93.6	47.6	99.4	59.3	91.0	94	48.0	99.0	59.4	91.0	93.7	47.6	99.4
Cyclophosphamide	32.4	32.1	98.1	92.3	66.7	15.3	15.4	98.2	85.7	61.1	70.0	79.5	89.6	84.9	85.6	69.0	79.5	89	83.8	85.7	76.0	87.1	88.9	85.0	90.6
Docetaxel	30.9	27.6	97.6	84.2	74.7	−0.8	1.7	97.6	25.0	68.1	68.4	83.0	86.5	74.2	92.4	66.0	83.0	86	73.1	91.6	70.4	88.1	85.8	74.3	94.0
Doxorubicin	42.3	31.6	100.0	100.0	85.0	24.4	18.0	99.3	87.5	82.0	78.2	82.0	96.0	84.0	95.2	79.0	82.5	96	84.2	95.3	84.0	89.7	95.9	85.4	97.2
Paclitaxel	33.7	27.3	98.0	75.0	86.1	34.1	26.5	98.7	81.8	85.7	61.0	67.0	93.4	69.0	93.0	62.0	67.6	93	69.7	92.8	66.2	73.5	93.4	71.4	94.0
**Endocrine therapy**																									
Tamoxifen	−1.7	0.0	99.3	0.0	74.5	34.0	33.3	94.9	69.6	80.4	60.0	78.7	85.4	65.0	92.1	60.0	81.2	83	62.9	92.7	56.9	81.2	81.9	60.9	92.6
Aromatase inhibitors^**c**^	3.70	3.0	100.0	100.0	63.9	19.5	25.0	91.6	62.9	68.5	45.0	79.8	69.5	59.1	85.4	47.0	81.0	70	60.1	86.5	47.1	81.0	69.8	60.7	86.2
**Biologic therapy**																									
Trastuzumab	−2.2	0.0	96.7	0.0	98.3	52.3	47.4	97.6	69.2	94.2	79.4	95.0	96.0	72.0	99.4	79.4	94.7	96	72.0	99.4	18.3	100.0	87.4	11.5	100.0

*Note:* One patient for carboplatin and two patients for doxorubicin had these treatments beyond the first course (postprimary treatment was not included in this analysis).

Abbreviations: SE, sensitivity; SP, specificity; PPV, positive predictive value; NPV, negative predictive value; ĸ, kappa statistic (values from 0.41 to 0.60 = moderate, 0.61 to 80 = substantial, and ≥ 0.81 = perfect agreement).

^**a**^ All sources included electronic medical records (EMR) from the University of Utah health pharmacy and Intermountain Healthcare, Utah statewide database (Inpatient Hospital Claims Utah, Ambulatory Surgery Utah, and Emergency Department Utah), Utah All‐Payer Claims Database (APCD), and pharmacy and medical claims provided by the Utah Cancer Registry (UCR).

^**b**^ Any chemotherapy, having received at least one chemotherapy agent.

^**c**^ Aromatase inhibitors (AIs) included anastrozole, letrozole, or exemestane.

Comparison between cumulative dose estimated for the five most commonly recommended chemotherapy agents from the APCD and the abstracted doses ranged from fair to good consistency between chemotherapy dose estimated from the claims and the abstraction (Table 4 and overlapping histograms presented in Figure S1 available online). The highest dose consistency between the two data sources was observed for doxorubicin (ICC = 75.0%, 95% CI 69.2, 81.5). Consistent with the primary analysis, the dose agreement between the abstraction and the claims did not change for cyclophosphamide or docetaxel, the sensitivity analysis showed similar ICC estimates when the dose calculations were not based on BSA. The ICC changed from 0.79 for doxorubicin and 0.72 for paclitaxel (data not shown) without BSA dose calculation (Table 4). The median radiotherapy cumulative dose was 50 Gray (range = 16–66) in the claims. The overall agreement (ĸ = 50.0%) was 50%, and the sensitivity and PPV were moderate between the claims and abstraction (Table 5). When assessed continuously, the ICC coefficient was moderate (ICC = 61.2%, 95% CI = 54.8, 71.9) for the cumulative radiation dose comparison in the two data sources, suggesting a moderate similarity between radiation dose and fractions estimated based on the identified information (fractions) in both data sources (Table 5).

**TABLE 4:** Comparison of chemotherapy dose between All‐Payer Claims Database (APCD)^a^ and abstraction (gold standard) among 186 patients diagnosed with breast cancer in 2013.

Chemotherapy agents^b^ (cumulative dose)	ICC (%)^c^	(95% CI)
Carboplatin, mg^d^	62.6	(53.9, 71.2)
Cyclophosphamide, mg/m^2^	69.2	(63.7, 77.9)
Docetaxel, mg/m^2^	68.8	(62.4, 77.1)
Paclitaxel, mg/m^2^	63.2	(55.6, 72.4)
Doxorubicin, mg/m^2^	75.8	(69.2, 81.5)

Abbreviations: CI, confidence interval; ICC, intraclass correlation coefficient.

^a^ APCD was combined with pharmacy and medical claims provided by the Utah Cancer Registry (UCR).

^b^ Chemotherapy dosing was estimated using National Comprehensive Cancer Network (NCCN) guidelines [16].

^c^ ICC was calculated to measure an agreement between APCD and abstraction dose for the five most commonly identified chemotherapy agents (values from 0.50 to 0.75 indicate moderate agreement, > 0.75 good agreement, and > 90 excellent agreement) [22].

^d^ Dose calculation was based on the patient's body surface area (BSA), as the recommended NCCN treatment regimens (mg/m^2^) were normalized to the BSA dosing. Calvert's formula was used for Carboplatin dosing, with estimated glomerular filtration rate for women: (GFR) = 0.85 * [(140—age) * weight (kg)/72 * (serum creatinine of 80, mg/dl)].

**TABLE 5:** Comparison of radiotherapy information between All‐Payer Claims Database (APCD)^a^ and abstraction (gold standard) among 186 patients diagnosed with breast cancer in 2013.

	Neither abstraction nor APCD		Abstraction only		APCD only		Both abstraction and APCD		ĸ		Sensitivity		Specificity		Positive predictive value		Negative predictive value	
Radiotherapy completion^c^	*N* (Row %)								%	(95% CI)	%	(95% CI)	%	(95% CI)	%	(95% CI)	%	(95% CI)
Complete	61	(37.6)	30	(18.5)	14	(8.6)	57	(35.2)	50.0	(39.0, 60.0)	65.5	(55.0, 75.5)	81.3	(72.5, 90.1)	80.3	(71.0, 89.5)	67.0	(57.4, 77.0)
Incomplete	167	(89.8)	—^b^	—^b^	—^b^	—^b^	—^b^	—^b^	5.0	(−13.8, 23.7)	9.1	(0.20, 41.3)	95.4	(91.0, 98.9)	12.5	(0.30, 53.0)	93.5	(88.4, 96.8)

Radiotherapy fractions	Mean (±SD)				Median			Range	ICC	(95% CI)								
Fractions (integers)		23.5		(9.6)	28.0			5–38	53.9	(48.2, 67.2)								
Cumulative dose (Gy)^d^		50.1		(14.5)	52.0			16–66	61.2	(54.8, 71.9)								

*Note:* Estimated dose per fraction in APCD: If 16 or fewer fractions were identified in the claims, but the number of fractions did not correspond to an NCCN standard (5, 10, and 15–16 fractions), 2.67 Gy per fraction was assigned. If 17–21 fractions were identified but the number of fractions did not correspond to an NCCN (15–16 fractions), 2.66 Gy per fraction for 15–16 fractions, followed by 2.50 Gy per fraction for the remaining fractions were assigned. If 22 or more fractions were identified in claims but the number of fractions did not correspond to an NCCN (25–28 fractions), 2 Gy per fraction was assigned. Within the abstracted data, radiotherapy information included the number of total fractions and total dose in gray (Gy) received for the initial radiotherapy regimens and the boost treatments. Abstracted radiotherapy values greater than 66 Gy were assigned the high‐end dosage of 66 Gy as per NCCN‐recommended radiation treatment dosing.

Abbreviations: CI, confidence interval; Gy, gGray; ĸ, kappa statistic; NPV, negative predictive value; PPV, positive predictive value; SD, standard deviation; SE, sensitivity; SP, specificity; (values from 0.41 to 0.60 = moderate, 0.61 to 0.80 = substantial, and ≥ 0.81 = perfect agreement); ICC, intraclass correlation coefficient, agreement assessed on cumulative dose calculation made comparable to that of abstraction total dose (< 0.50 weak, 0.50 to 0.75 moderate, and > 0.75 indicates good agreement).

^a^ APCD was combined with pharmacy and medical claims provided by the Utah Cancer Registry (UCR).

^b^ Suppressed if the number of observations was < 11 in accordance with the data confidentiality policy.

^c^ Rows do not add up to 100% due to missing observations (there were 23 missing observations in the abstraction and 1 observation in APCD).

^d^ APCD radiotherapy dose and completion were determined based on National Comprehensive Cancer Network (NCCN) guidelines [16].

## 4 Discussion

This pilot study assessed the validity of the Utah APCD in identifying commonly prescribed breast cancer chemotherapy agents, their related dosage, and radiotherapy specific information against the gold‐standard abstraction. Medical records abstraction is costly and time‐consuming; to our knowledge, this is the first publication to show the validity of the APCD to identify specific breast cancer treatment agents and dosage against the medical abstractions. The overall utility of the APCD in identifying overall chemotherapy, namely carboplatin and trastuzumab, was high, with some variation in sensitivity across other treatment agents. Consistent with prior SEER‐Medicare studies focusing on radiotherapy receipt, [3, 6] moderate radiotherapy completion and dose consistency between claims and the gold standard was observed in this pilot study. Similarly, moderate consistency was observed in estimating chemotherapy dose.

A similar study that evaluated the completeness of breast cancer chemotherapy agents in the claims by Warren et al. compared SEER Patterns of Care (POC) studies to Medicare claims for breast cancer patients 65 years of age or older from 1991 through 1995 [6]. The higher sensitivity in this study for cyclophosphamide may be in part due to the addition of private insurance in APCDs and improved billing codes for chemotherapy treatments from two decades ago. The recognized limitation of cancer registries in capturing complete treatment data, particularly concerning breast cancer treatments such as chemotherapy, radiation therapy, and hormone therapy, has been acknowledged [3]. However, the current comparisons of APCDs to respective SEER cancer registries, [23, 24] also in the absence of chemotherapy agent names or dosage and radiotherapy specific information presented, do not inherently validate the claims.

In this study, the majority of patients who received chemotherapy had the NCCN‐recommended number of cycles identified in the claims, suggesting the APCD's potential to address the well‐known treatment incompleteness in cancer registries. Patients receiving more treatments than recommended by the NCCN may have had metastatic breast cancer that generally may require more treatment. Similarly, fewer treatments identified than recommended by the NCCN may be due to incompleteness of the APCD or treatment toxicities requiring premature discontinuation of treatment for some patients. In addition, the lower sensitivity for paclitaxel and cyclophosphamide (67.6% and 79.5%) in this study may be a result of cancer treatments being administered outside of the hospital setting, such as in outpatient care facilities that may be captured by the APCD but more likely to be missed by the cancer registry. However, the abstractor discovered four additional patients treated with cyclophosphamide and one fewer patient treated with paclitaxel than what was reported in the claims for these chemotherapy agents. Regarding biologic and endocrine therapy, high sensitivity of the APCD to identify trastuzumab and endocrine therapy was observed using these methods.

To our knowledge, this study is the first to assess the validity of the APCD in identifying specific chemotherapy agents, dosage, and radiotherapy completion and dosage against the abstraction. In addition to public payers, the APCD encompasses the majority of claims from various health insurance carriers, and third‐party administrators, inpatient and outpatient providers, pharmacy claims, medical claims, and dental claims across care sites, extending beyond hospitalizations and emergency department visits. Thus, this study demonstrated that APCD captured a high proportion of the most common treatments received by breast cancer patients, particularly when compared to EMR, SHFD, and other medical records from the UCR. Other states with access to APCDs can benefit from using these methods to leverage the claims for identifying patients receiving specific breast cancer treatment without the need for data abstraction.

The primary inherent limitation of this pilot study is the small sample size. However, while otherwise rare, the availability of abstraction records, allowed for the validation of the APCD in identifying breast cancer‐specific treatment information. Given the recognized incompleteness of cancer treatment information in cancer registries, the abstraction of medical records was completed by certified CTRs on a subset of most completed records available in the registry, hence serving as the gold‐standard for validation of data sources such as APCDs. It is still possible that some treatments may have been unavailable in the registry at the time when the data were abstracted, especially endocrine therapy use, which can be difficult to identify in cancer registries. Overall, claims data and cancer registries have different structures and methods of data collection, which can lead to discrepancies in the treatments they capture. The claims do not cover 100% of the population and are driven by timely billing and insurance requirements. In contrast, cancer registries are a comprehensive source of all aspects of cancer care, including treatments that are used for research and public health purposes. Lastly, to ensure the inclusion of a comprehensive list of oncology medications, we utilized the regularly updated CanMED‐HCPCS database for identifying chemotherapy, endocrine therapy, and trastuzumab [11]. While it is possible for some billing codes to be discontinued over time, we did not identify any codes in the claims that had been discontinued during the study period.

The misclassification of patients with chemotherapy or/and radiotherapy (false positives) is expected to be minimal, as those receiving chemotherapy are identified through distinct billing codes that also have corresponding distinct treatment dates in the claims. Additionally, the number of treatment cycles or doses recorded in the registry is cross‐checked by a certified tumor registrar, making it unlikely for these details to be present for patients who did not receive any cancer treatments. However, for patients identified as not having received either treatment (false negatives), it is likely that at least one of the data sources would have captured these patients. If these patients were not captured by either source, it would not affect the agreement observe between the two data sources. For advanced‐stage cancer, patients may receive more treatment and may have more misclassification. We did not capture patients not covered by health insurance or patients covered with other insurance such as the Veteran Health Administration. This study also did not abstract data for older patients because the initial assumption was that the majority of patients aged ≥ 65 would be insured by Medicare. However, it is noteworthy that APCD was also useful for the older cancer patient populations [10].

In conclusion, by employing these novel methods, we demonstrated the high reliability of claims in identifying most breast cancer treatment agents overall, namely carboplatin, docetaxel, and trastuzumab. Moderate dose consistency in the claims compared with the gold‐standard abstraction was observed using these methods. The claims, in combination with multiple data sources, improved the completeness of cancer treatment information. APCDs have the potential to significantly augment cancer registry data, specifically considering that cancer registries do not currently provide chemotherapy agent names or dosages for research.

## Author Contributions

**Alzina Koric:** formal analysis (lead), methodology (equal), writing – original draft (lead), writing – review and editing (lead). **Chun‐Pin Esther Chang:** conceptualization (equal), project administration (equal), writing – review and editing (equal). **Shane Lloyd:** methodology (equal), writing – review and editing (equal). **Mark Dodson:** project administration (equal). **Vikrant G. Deshmukh:** data curation (equal). **Michael G. Newman:** data curation (equal). **Ankita P. Date:** data curation (equal). **Jen A. Doherty:** conceptualization (equal), funding acquisition (equal), project administration (equal). **Lisa H. Gren:** writing – review and editing (equal). **Christina A. Porucznik:** writing – review and editing (equal). **Benjamin A. Haaland:** writing – review and editing (equal). **N. Lynn Henry:** methodology (equal), writing – review and editing (equal). **Mia Hashibe:** conceptualization (equal), project administration (lead), resources (lead), software (lead), supervision (lead), writing – review and editing (equal).

## Conflicts of Interest

The authors declare no conflicts of interest.

## Supporting information


Figure S1.
Figure S2.

Table S1.
Table S2.
Table S3.
Table S4.
Table S5.

## Data Availability

Data used for this study can be accessed through the approval of the Resource for Genetic and Epidemiologic Research Committee (RGE), the oversight committee for the UPDB and IRB.
