# Supplementary material for: Capturing Chemotherapy and Radiotherapy Dose Among Breast Cancer Patients With the Utah All‐Payer Claims Database Compared With Gold‐Standard Abstraction
Source: Cancer Med. 2024 Nov 15;13(22):e70411. doi: 10.1002/cam4.70411 (PMC11568241; doi:10.1002/cam4.70411)
Supplement: Supplementary file 1 — Figure S1. Figure S2. [file CAM4-13-e70411-s001.docx]

| **SUPPLEMNTARY FIGURE S1**. Overalapping histograms for chemotherapy dose in APCD and abstraction | |
| --- | --- |
| 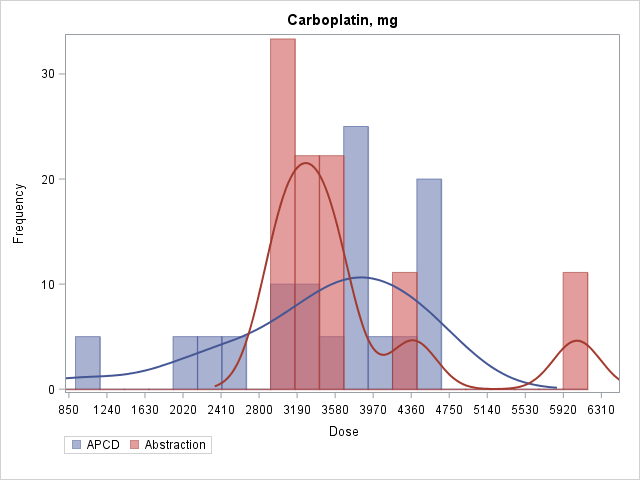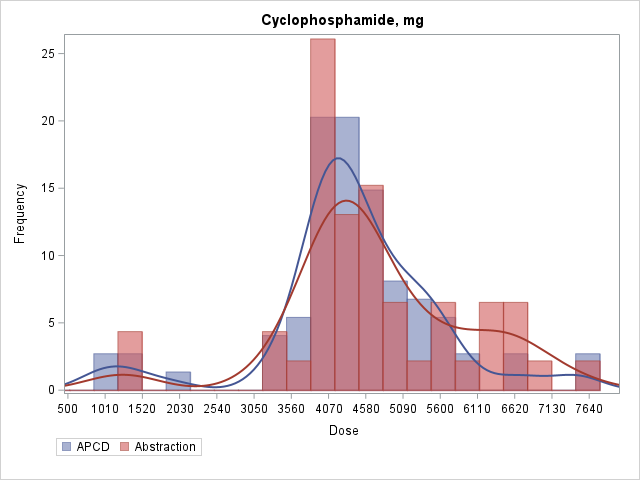 | 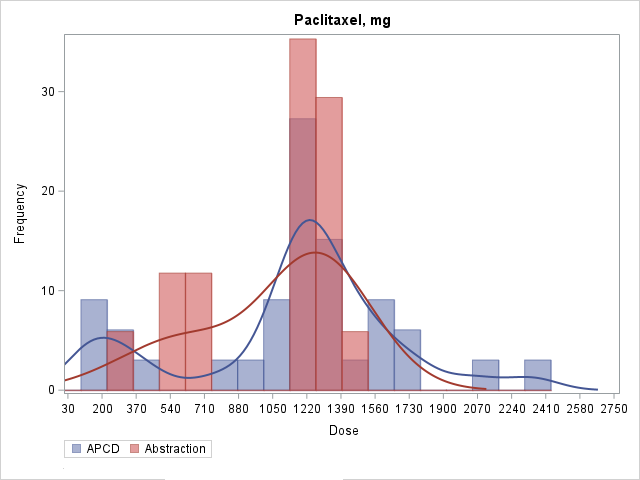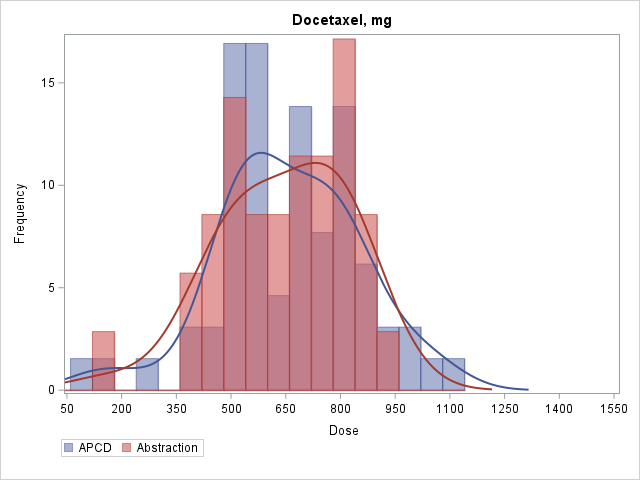 |
| 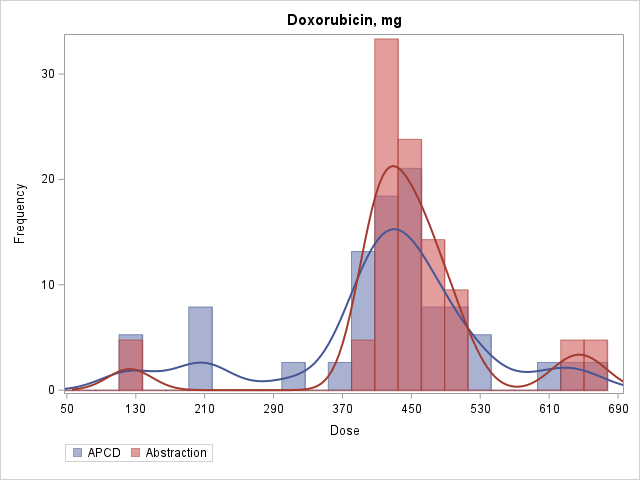 |  |

| **SUPPLEMENTARY FIGURE S2**. Overalapping histograms for radiotherpay dose and number of radiotherapy fractions (integers) in APCD and abstraction | |
| --- | --- |
| 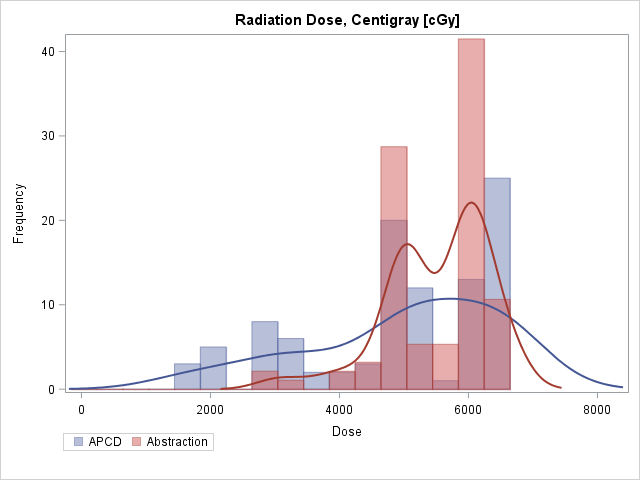 | 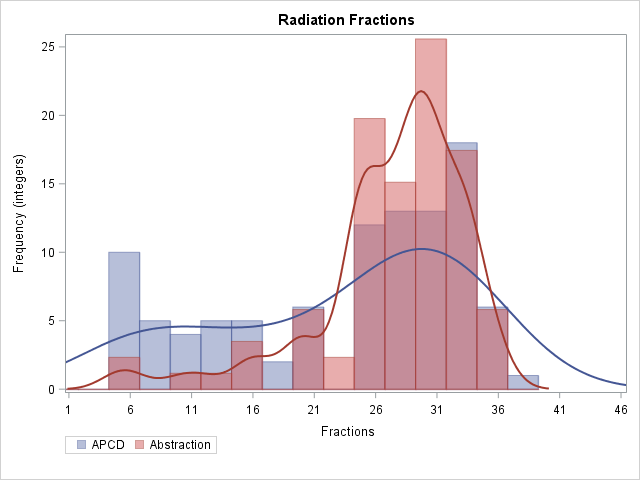 |
