# Supplementary material for: Capturing Chemotherapy and Radiotherapy Dose Among Breast Cancer Patients With the Utah All‐Payer Claims Database Compared With Gold‐Standard Abstraction
Source: Cancer Med. 2024 Nov 15;13(22):e70411. doi: 10.1002/cam4.70411 (PMC11568241; doi:10.1002/cam4.70411)
Supplement: Supplementary file 2 — Table S1. Table S2. Table S3. Table S4. Table S5. [file CAM4-13-e70411-s002.docx]

| **SUPPLEMENTARY TABLE S1.** Example calculation for chemotherapy dosing in APCD and abstraction (gold-standard) | |
| --- | --- |
| Chemotherapy agents identified based on HCPCS/CPT billing codes | NCCN preferred recommended dose and duration for adjuvant therapy **^b^** |
| I.     Carboplatin, mg | Carboplatin (mg) = Area Under the Curve (AUC) 6 * (GFR + 25) **^a^** |
| II.   Cyclophosphamide, mg/m^2^ | 600 mg/m^2^ IV, cycled every 14 or 21 days for 4 or 6 cycles |
| III.  Docetaxel, mg/m^2^ | 75 mg/m^2^ IV, cycled every 21 days for 4 or 6 cycles |
| IV.  Paclitaxel, mg/m^2^ | 80 or 175 mg/m^2^ IV, cycled weekly for 12 weeks/every 14 days for 4 cycles, respectively |
| V.   Doxorubicin, mg/m^2^ | 60 mg/m^2^ IV, cycled every 14 days or 21 days for 4 cycles |
| Abbreviations: HCPCS/CPT, Healthcare Common Procedure Coding System/Current Procedural Terminology.  Dose calculation was based on patient’s body surface area (BSA), as these recommended NCCN treatment regimens (mg/m^2^) were normalized to the BSA dosing.  ^a^ Calvert's formula for Carboplatin dosing calculation, with expected average glomerular filtration rate for women:  (GFR) = 0.85 * [ (140 - Age) * Weight (kg) / 72 * (Serum Creatinine of 80) ].  **^b^** Other than the preferred recommended dose was assigned if patient received other recommended regimens, such as: Cyclophosphamide: EC (Epirubicin + Cyclophosphamide), 830 mg/m2 IV, cycled every 21 days for 8 cycles. Docetaxel: AC (Doxorubicin + Cyclophosphamide), with Docetaxel + Trastuzumab, 100 mg/m2 IV, every 21 days for 4 cycles. Doxorubicin: TAC (Docetaxel + Doxorubicin + Cyclophosphamide), 50 mg/m2 IV, cycled every 21 days for 6 cycles. | |

| **SUPPLEMENTARY TABLE S2.** Example calculation for radiotherapy dosing and completion in APCD (radiotherapy dose was provided in abstraction) | | | |
| --- | --- | --- | --- |
| Radiotherapy dosing | |  | Radiotherapy  completed: |
| Number of fractions identified in APCD based on CPT billing codes | Recommended NCCN  dose, Gray (Gy) / fractions (+ boost) | Total dose, Centi-gray (cGy) |  |
| 5 | 30 Gy / 5 | 3000 | Yes |
| 6 | 2.67 Gy | 1602 **^a^** | Yes |
| 7 | 2.67 Gy | 1869 **^a^** | Yes |
| 8 | 2.67 Gy | 2136 | No **^e^** |
| 10 | 34 Gy / 10 or 38.5 Gy / 10 | 3400 | Yes |
| 11 | 2.67 Gy | 2937 **^a^** | Yes |
| 12 | 2.67 Gy | 3024 **^a^** | Yes |
| 14 | 2.67 Gy | 3738 **^a^** | Yes |
| 15 | 40-42.5 Gy / 15-16 (+10-16 Gy / 4-8) | 4050 | Yes |
| 16 | 40-42.5 Gy / 15-16 (+10-16 Gy / 4-8) | 4320 | Yes |
| 18 | 2.66 Gy / 15 (+2.50 Gy) | 5300 **^b^** | Yes |
| 19 | 2.66 Gy / 15 (+2.50 Gy) | 5300 **^b^** | Yes |
| 20 | 2.66 Gy / 15 (+2.50 Gy) | 5300 **^b^** | Yes |
| 25 | 45-50.4 Gy / 25-28 (+10-16 Gy / 4-8) | 5000 | Yes |
| 26 | 45-50.4 Gy / 25-28 (+10-16 Gy / 4-8) | 5200 **^c^** | No **^e^** |
| 27 | 45-50.4Gy / 25-28 (+10-16 Gy / 4-8) | 5400 **^c^** | No **^e^** |
| 28 | 45-50.4Gy / 25-28 (+10-16 Gy / 4-8) | 5040 **^c^** | Yes |
| 29 | 45-50.4 Gy / 25-28 (+10-16 Gy / 4-8) | 5800 **^c^** | Yes |
| 30 | 45-50.4 Gy / 25-28 (+10-16 Gy / 4-8) | 6000 **^c^** | Yes |
| 33 | 45-50.4 Gy / 25-28 (+10-16 Gy / 4-8) | 6600 **^c^** | Yes |
| 34 | 45-50.4 Gy / 25-28 (+10-16 Gy / 4-8) | 6600 **^d^** | Yes |
| 35 | 45-50.4 Gy / 25-28 (+10-16 Gy / 4-8) | 6600 **^d^** | Yes |
| 38 | 45-50.4 Gy / 25-28 (+10-16 Gy / 4-8) | 6600 **^d^** | Yes |
| Abbreviations: NCCN, National Comprehensive Cancer Network guidelines in oncology; CPT, Current Procedural Terminology.  **^a^** If 16 or fewer fractions identified in APCD, but the number of fractions does not correspond to an NCCN standard (5, 10, and 15-16 fractions), assumed were 2.67 Gy per fraction.  **^b^** If 17-21 fractions identified in APCD, but the number of fractions does not correspond to an NCCN standard (15-16 fractions), assumed were 2.66 Gy per fraction for 15-16 fractions, followed by 2.50 Gy per fraction for the remaining fractions. **^c^** If 22+ fractions identified in APCD, but the number of fractions does not correspond to an NCCN standard (25-28 fractions), assumed were 2 Gy per fraction, and considered a completed treatment, given high dose identified. **^d^** Maximum dose of 66 Gy was assigned for these patients identified in the claims.  **^e^** Most likely incomplete, unable to make a reasonable assumption in the claims. | | | |

| **SUPPLEMENTARY TABLE S3.** Comparison of cancer treatment information identified in pharmacy and medical claims ^a^ and abstraction (gold-standard) among 186 breast cancer patients diagnosed in 2013**, with 95%CI** | | | | | | | | | | |
| --- | --- | --- | --- | --- | --- | --- | --- | --- | --- | --- |
|  | Pharmacy and medical claims from UCR | | | | | | | | | |
| Treatment agents | ĸ | | Sensitivity | | Specificity | | Positive  predictive value | | Negative  predictive value | |
|  | % | (95%CI) | % | (95%CI) | % | (95%CI) | % | (95%CI) | % | (95%CI) |
| Chemotherapy, any **^b^** | 26.0 | (16.6, 35.4) | **30.8** | (22.1, 40.6) | 97.6 | (91.5, 99.7) | **94.1** | (80.0, 99.3) | 52.6 | (44.4, 60.8) |
| **CHEMOTHERAPY AGENTS** |  |  |  |  |  |  |  |  |  |  |
| Carboplatin | 28.0 | (0.07, 55.9) | **27.3** | (6.0, 61.9) | 97.1 | (93.5, 99.1) | **37.5** | (8.50, 75.5) | 95.5 | (91.3, 98.0) |
| Cyclophosphamide | 15.3 | (5.8, 24.7) | **15.4** | (8.2, 25.3) | 98.2 | (93.5, 99.8) | **85.7** | (57.2, 98.2) | 61.1 | (53.9, 68.9) |
| Docetaxel | –0.80 | (-6.5, 4.70) | **1.70** | (0.04, 9.0) | 97.6 | (93.2, 99.5) | **25.0** | (0.60, 80.6) | 68.1 | (60.8, 74.8) |
| Doxorubicin | 24.4 | (8.7, 40.1) | **18.0** | (7.5, 33.5) | 99.3 | (96.3, 100.0) | **87.5** | (47.3, 99.7) | 82.0 | (75.6, 87.4) |
| Paclitaxel | 34.1 | (16.2, 52.0) | **26.5** | (12.9, 44.4) | 98.7 | (95.3, 99.8) | **81.8** | (48.2, 97.7) | 85.7 | (79.6, 90.5) |
| **ENDOCRINE THERAPY** |  |  |  |  |  |  |  |  |  |  |
| Tamoxifen | 34.0 | (18.6, 49.5) | **33.3** | (20.4, 48.4) | 94.9 | (89.8, 97.9) | **69.6** | (47.1, 86.8) | 80.4 | (73.4, 86.2) |
| Aromatase inhibitors **^c^** | 19.5 | (6.5, 32.6) | **25.4** | (15.5, 37.5) | 91.6 | (85.1, 95.9) | **62.9** | (42.4, 80.6) | 68.5 | (60.7, 75.7) |
| **BIOLOGIC THERAPY** |  |  |  |  |  |  |  |  |  |  |
| Trastuzumab | 52.3 | (30.5, 74.1) | **47.4** | (24.4, 71.1) | 97.6 | (94.0, 99.3) | **69.2** | (38.6, 90.1) | 94.2 | (89.6, 97.2) |
| Abbreviations: ĸ, kappa statistic was used to measure agreement (values from 0.41 to 0.60 indicate moderate, 0.61 to 80 indicate substantial, and ≥0.81 indicate perfect agreement); CI, confidence intervals. ^a^ Pharmacy and medical claims provided by the Utah Cancer Registry (UCR).  ^b^ Any chemotherapy, having received at least one chemotherapy. ^c^ Aromatase inhibitors included anastrozole, letrozole, or exemestane. | | | | | | | | | | |

| **SUPPLEMENTARY TABLE S4.** Comparison of cancer treatment information identified in electronic medical records **^a^** and abstraction (gold-standard) among 186 breast cancer patients diagnosed in 2013**,** **with 95%CI** | | | | | | | | | | | |
| --- | --- | --- | --- | --- | --- | --- | --- | --- | --- | --- | --- |
|  | Electronic medical records (EMRs) | | | | | | | | | | |
| Treatment agents | ĸ | | Sensitivity | | Specificity | | Positive  predictive value | | Negative  predictive value | | |
|  | % | 95%CI | % | 95%CI | % | 95%CI | % | 95%CI | % |  | 95%CI |
| Chemotherapy, any **^b^** | 24.5 | (15.2, 33.8) | **29.1** | (20.6, 38.9) | 97.6 | (91.5, 1.00) | **93.7** | (79.2, 99.2) | 52.3 | (44.1, 60.4) | |
| **CHEMOTHERAPY AGENTS** |  |  |  |  |  |  |  |  |  |  | |
| Carboplatin | 51.8 | (21.1, 82.5) | **36.4** | (10.9, 69.2) | 100.0 | (97.9, 100.0) | **100.0** | (39.8, 100.0) | 96.1 | (92.2, 98.4) | |
| Cyclophosphamide | 32.4 | (21.8, 44.0) | **32.1** | (21.1, 42.7) | 98.1 | (93.5, 99.8) | **92.3** | (75.0, 99.0) | 66.7 | (58.8, 73.9) | |
| Docetaxel | 30.9 | (17.3, 44.4) | **27.6** | (16.6, 40.9) | 97.6 | (93.2, 99.5) | **84.2** | (60.4, 96.6) | 74.7 | (67.4, 81.1) | |
| Doxorubicin | 42.3 | (25.5, 59.1) | **31.6** | (17.5, 48.6) | 100.0 | (97.5, 100.0) | **100.0** | (73.5, 100.0) | 85.0 | (78.8, 89.9) | |
| Paclitaxel | 33.7 | (15.5, 51.9) | **27.3** | (13.3, 45.5) | 98.0 | (94.3, 99.5) | **75.0** | (42.8, 94.5) | 86.1 | (80.1, 90.9) | |
| **ENDOCRINE THERAPY** |  |  |  |  |  |  |  |  |  |  | |
| Tamoxifen | -1.7 | (-3.1, 1.0) | **0.0** | (0.0, 8.0) | 99.3 | (96.0, 99.8) | **0.0** | (0.0, 97.5) | 74.5 | (67.5, 80.6) | |
| Aromatase inhibitors **^c^** | 3.7 | (-1.3, 8.8) | **3.0** | (4.0, 10.4) | 100.0 | (96.8, 100.0) | 100.0 | (15.8, 100.0) | 63.9 | (56.4, 70.9) | |
| **BIOLOGIC THERAPY** |  |  |  |  |  |  |  |  |  |  | |
| Trastuzumab | -2.2 | (-3.9, -4.3) | **0.0** | (0.0, 71.0) | 96.7 | (93.0, 98.8) | **0.0** | (0.0, 45.9) | 98.3 | (95.2, 99.6) | |
| Abbreviations: ĸ, kappa statistic was used to measure agreement (values from 0.41 to 0.60 indicate moderate, 0.61 to 80 indicate substantial, and ≥0.81 indicate perfect agreement); CI, confidence intervals. ^a^ Electronic Medical Records (EMR) from University of Utah health pharmacy and Intermountain Healthcare and Utah statewide database (Inpatient Hospital Claims Utah, Ambulatory Surgery Utah, and Emergency Department Utah). ^b^ Any chemotherapy, having received at least one chemotherapy. ^c^ Aromatase inhibitors included anastrozole, letrozole, or exemestane. | | | | | | | | | | | |

| **SUPPLEMENTARY TABLE S5.** Comparison of cancer treatment information identified in all sources ^a^ and abstraction (gold-standard) among 186 breast cancer patients diagnosed in 2013, **with 95%CI** | | | | | | | | | | |
| --- | --- | --- | --- | --- | --- | --- | --- | --- | --- | --- |
|  | All Sources | | | | | | | | | |
| Treatment agents | ĸ | | Sensitivity | | Specificity | | Positive  predictive value | | Negative  predictive value | |
|  | % | 95%CI | % | 95%CI | % | 95%CI | % | 95%CI | % | 95%CI |
| Chemotherapy, any **^b^** | 87.0 | (80.0, 94.0) | **96.1** | (90.4, 98.9) | 90.2 | (81.7, 95.7) | **92.6** | (85.9, 96.7) | 94.9 | (87.4, 98.6) |
| **CHEMOTHERAPY AGENTS** |  |  |  |  |  |  |  |  |  |  |
| Carboplatin | 59.4 | (39.0, 79.8) | **91.0** | (58.7, 99.0) | 93.7 | (89.0, 96.8) | **47.6** | (25.7, 70.2) | 99.4 | (97.0, 100.0) |
| Cyclophosphamide | 76.0 | (66.3, 85.3) | **87.1** | (77.6, 93.7) | 88.9 | (81.4, 94.1) | **85.0** | (75.3, 92.0) | 90.6 | (83.3, 95.4) |
| Docetaxel | 70.4 | (59.8, 81.1) | **88.1** | (77.1, 95.1) | 85.8 | (78.5, 91.4) | **74.3** | (62.4, 84.0) | 94.0 | (88.0, 87.5) |
| Doxorubicin | 84.0 | (74.5, 93.6) | **89.7** | (75.8, 97.1) | 95.9 | (91.3, 98.5) | **85.4** | (70.8, 94.4) | 97.2 | (93.1, 99.2) |
| Paclitaxel | 66.2 | (52.2, 80.2) | **73.5** | (55.6, 87.1) | 93.4 | (88.2, 96.8) | **71.4** | (53.7, 85.4) | 94.0 | (90.0, 97.2) |
| **ENDOCRINE THERAPY** |  |  |  |  |  |  |  |  |  |  |
| Tamoxifen | 56.9 | (44.1, 69.6) | **81.2** | (67.4, 91.0) | 81.9 | (74.4, 87.9) | **60.9** | (47.9, 72.9) | 92.6 | (86.5, 96.6) |
| Aromatase inhibitors **^c^** | 47.1 | (34.7, 59.6) | **81.0** | (69.0, 89.2) | 69.8 | (60.1, 78.0) | **60.7** | (49.7, 70.9) | 86.2 | (77.5, 92.4) |
| **BIOLOGIC THERAPY** |  |  |  |  |  |  |  |  |  |  |
| Trastuzumab | 18.3 | (3.0, 36.4) | **100.0** | (29.0, 100.0) | 87.4 | (81.7, 91.9) | **11.5** | (2.4, 30.1) | 100.0 | (97.7, 100.0) |
| Abbreviations: ĸ, kappa statistic was used to measure agreement (values from 0.41 to 0.60 indicate moderate, 0.61 to 80 indicate substantial, and ≥0.81 indicate perfect agreement); CI, confidence intervals. ^a^ All sources included electronic medical records (EMRs) from University of Utah Health Pharmacy and Intermountain Healthcare, Utah statewide database (Inpatient Hospital Claims Utah, Ambulatory Surgery Utah, and Emergency Department Utah), Utah All-Payer Claims Database (APCD), and pharmacy and medical claims provided by Utah Cancer Registry (UCR). ^b^ Any chemotherapy, having received at least one chemotherapy. ^c^ Aromatase inhibitors included either anastrozole, letrozole, or exemestane. | | | | | | | | | | |
